# Supplementary figures and images for: Confounds of using the unc-58 selection marker highlights the importance of genotyping co-CRISPR genes
Source: PLoS One. 2022 Jan 18;17(1):e0253351. doi: 10.1371/journal.pone.0253351 (PMC8765651; doi:10.1371/journal.pone.0253351)

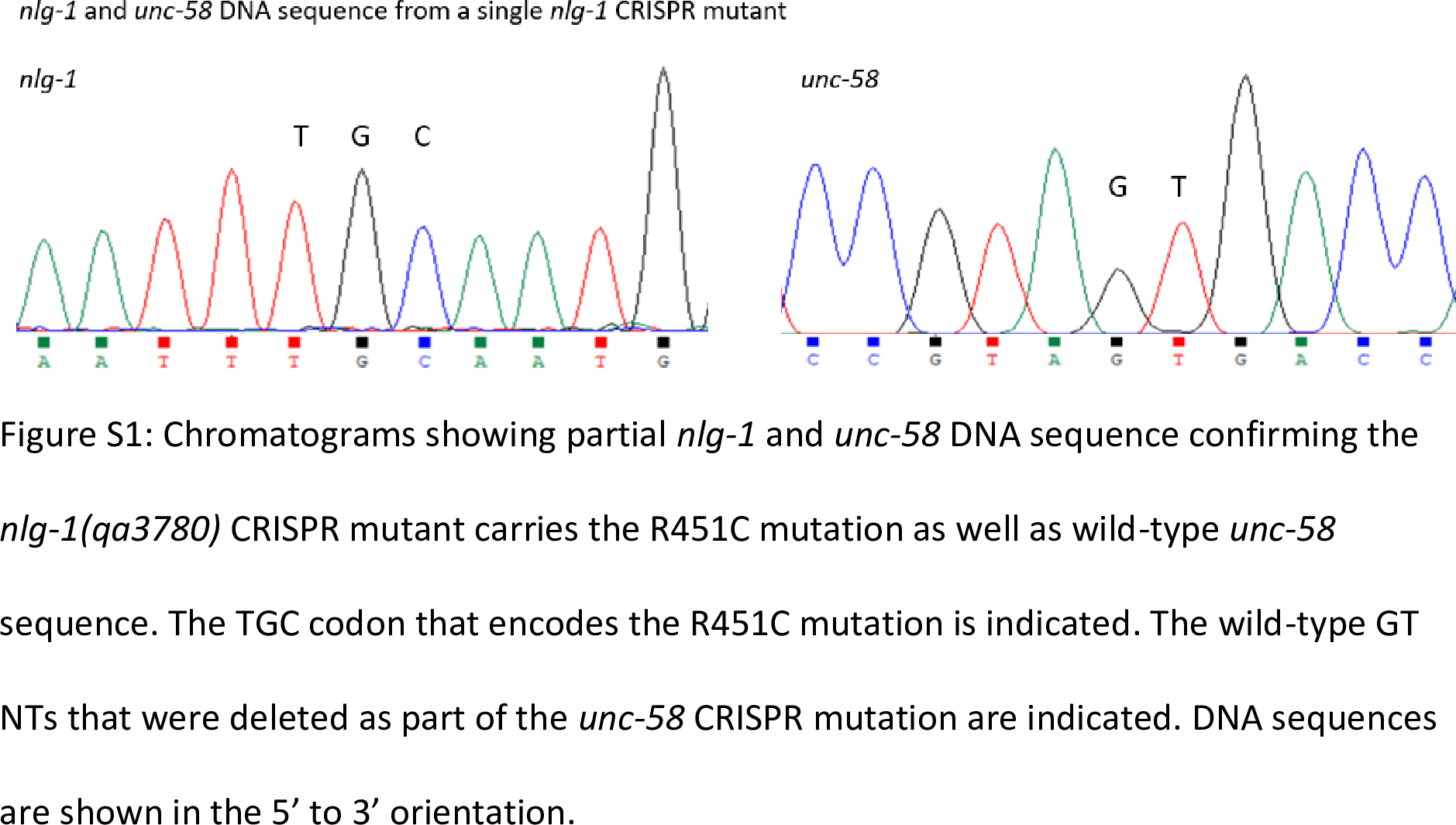

Supplement: S1 Fig — The TGC codon that encodes the R451C mutation is indicated. The wild-type GT NTs that were deleted as part of the unc-58 CRISPR mutation are indicated. DNA sequences are shown in the 5’ to 3’ orientation. (TIF) [file pone.0253351.s001.tif]
